# An Unusual Case of Obstructive Shock

**DOI:** 10.1016/j.jaccas.2021.10.017

**Published:** 2021-12-15

**Authors:** Simon Parlow, Matthew Cheung, Louis Verreault-Julien, Kai Yi Wu, Philip Berardi, Vidhya Nair, Pietro Di Santo, Richard G. Jung, Rebecca Mathew, Benjamin Hibbert

**Affiliations:** a CAPITAL Research Group, University of Ottawa Heart Institute, Ottawa, Ontario, Canada; b Division of Cardiology, University of Ottawa Heart Institute, Ottawa, Ontario, Canada; c Department of Medicine, University of Alberta, Edmonton, Alberta, Canada; d Department of Pathology and Laboratory Medicine, University of Ottawa and Ottawa Hospital Research Institute, The Ottawa Hospital, Ottawa, Ontario, Canada; e School of Epidemiology and Public Health, University of Ottawa, Ottawa, Ontario, Canada; f Faculty of Medicine, University of Ottawa, Ottawa, Ontario, Canada; g Department of Cellular and Molecular Medicine, University of Ottawa, Ottawa, Ontario, Canada

**Keywords:** cancer, cardiac tumor, echocardiography, hemodynamics, obstructive shock, right-sided catheterization, DLBCL, diffuse large B-cell lymphoma, IVC, inferior vena cava, LV, left ventricular, RA, right atrial, RV, right ventricular, SVC, superior vena cava, TV, tricuspid valve

## Abstract

A 54-year-old man presented in profound obstructive shock. Investigations revealed a right atrial mass causing severe right ventricular inflow obstruction and compromised cardiac output. The patient was treated with emergency balloon catheter intervention to relieve the obstruction, with resulting hemodynamic stability. The pathology report later returned a positive result for diffuse large B-cell lymphoma. (**Level of Difficulty: Intermediate.**)

## Graphical abstract

**Figure:**
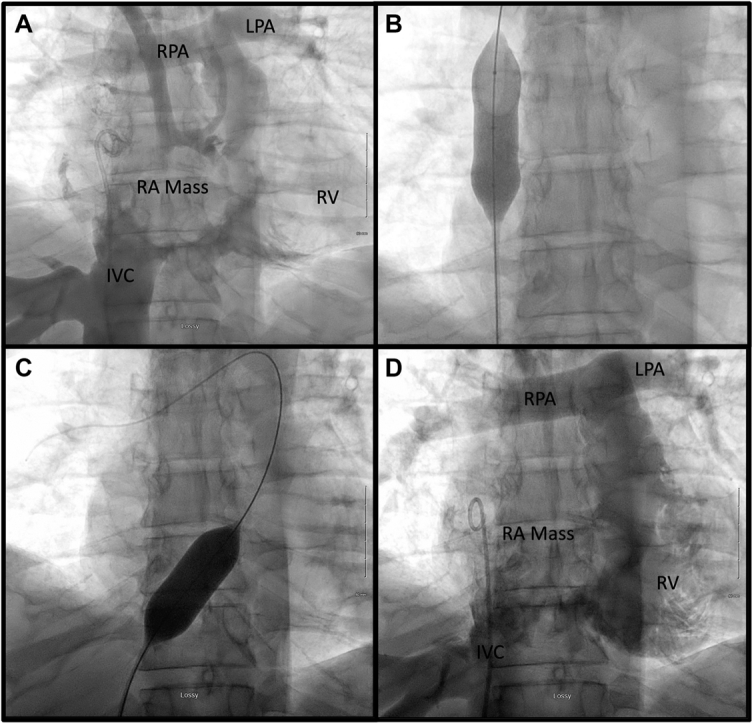

## History of Presentation

A 54-year-old man presented to a peripheral hospital with a 2-week history of dyspnea, leg swelling, abdominal bloating, and fatigue. Over the next 1 to 2 hours, the patient had progressive hypotension and drowsiness requiring intubation and initiation of vasopressor support. He was subsequently transferred to our quaternary care cardiac intensive care unit (University of Ottawa Heart Institute, Ottawa, Ontario, Canada) for further work-up and management.

**Learning Objectives**

• To be able to create a differential diagnosis for the patient presenting with obstructive shock.
• To understand the role of hemodynamics in the management of obstructive shock related to an intracardiac mass.

On arrival, he was profoundly hemodynamically unstable. He required 3 vasopressors to maintain a blood pressure of 75/58 mm Hg. He was bradycardic, with a heart rate of 47 beats/min in sinus rhythm. On physical examination, he was sedated and ventilated. He was mottled, with thready peripheral pulses and cool extremities. His neck and face were markedly discolored and swollen, with distended veins. His heart sounds were barely audible on precordial auscultation, with no murmurs or extra heart sounds heard. His lungs were clear to auscultation bilaterally. His abdomen was slightly distended but nonperitonitic. He had a Foley catheter inserted but had not produced urine for several hours. He was hypoxemic, with an arterial Po_2_ of 75 mm Hg despite ventilation with 100% inhaled fraction of inspired oxygen. His serum lactate level was 21.2 mmol/L, with an arterial pH of 6.85 and a serum bicarbonate level of 4 mmol/L.

## Past Medical History

Two weeks before his presentation, he had received a diagnosis of pericarditis during a visit to a peripheral emergency department and was started on colchicine and ibuprofen. His past medical history was otherwise significant only for cigarette smoking, with no previous home medications. He had no known allergies or illicit drug use.

## Differential Diagnosis

The patient’s clinical examination and biochemical findings are consistent with profound shock, marked by hypotension and clear evidence of end-organ hypoperfusion (decreased level of consciousness, anuria, and elevated lactate). There are several clinical signs that can be observed at the bedside to help elucidate the subtype of shock. In both cardiogenic and obstructive shock, the patient typically displays signs of low cardiac output such as cool extremities and thready pulses, as well as signs of high left ventricular (LV) or right ventricular (RV) filling pressures such as pulmonary edema, peripheral edema, and jugular venous distention (1). The combination of these clinical findings rules out other causes of shock such as hypovolemic and distributive, including septic shock. Furthermore, there were no obvious signs of infection. On the basis of our patient’s clinical examination, the differential diagnosis included obstructive shock from cardiac tamponade, massive pulmonary embolism or an obstructive mass, or cardiogenic shock resulting from severe LV or RV dysfunction.

## Investigations

We first performed an urgent point-of-care ultrasound examination to help elucidate the cause of the patient’s shock and observed a large mass in the patient’s right atrium with extension across the tricuspid valve (TV). RV inflow was clearly compromised, and the right ventricle was underfilled, resulting in reduced LV preload and cardiac output. The inferior vena cava (IVC) was plethoric and noncollapsible. A rapid bolus of intravenous crystalloid fluid was initiated, and the patient was taken on an emergency basis to the catheterization laboratory for further management.

**Figure 1:**
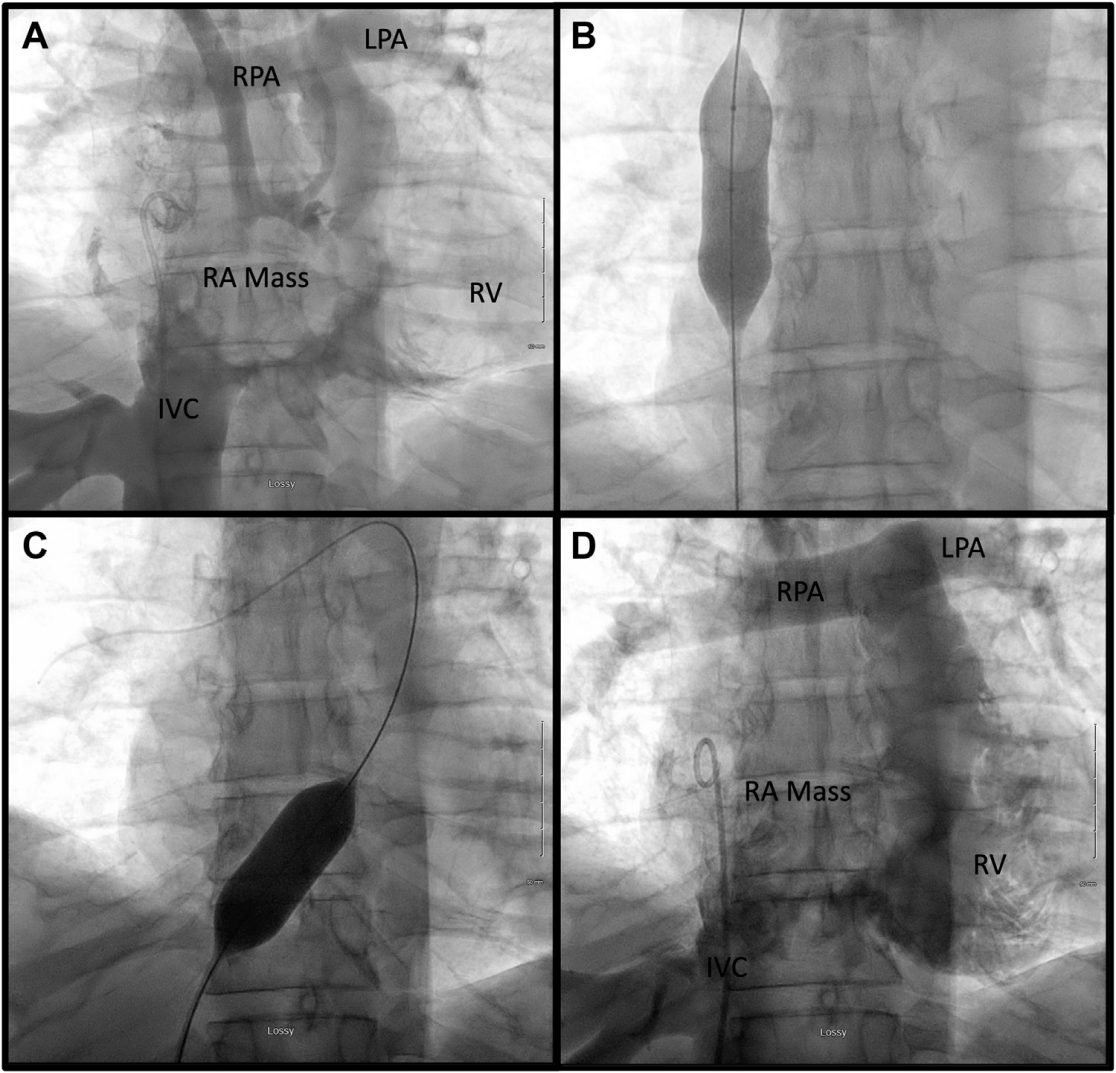
Percutaneous Catheter Intervention **(A)** Angiographic imaging showing a large filling defect in the right atrium (RA) consistent with a large mass and resulting in significant inflow restriction in the right ventricle (RV). **(B)** Targeted balloon intervention to the superior vena cava and **(C)** tricuspid valve annulus, resulting in **(D)** improvement in right ventricular filling. IVC = inferior vena cava; LPA = left pulmonary artery; RPA = right pulmonary artery.

We then performed a right-sided heart catheterization for hemodynamic assessment by navigating around the obstructive mass and into the pulmonary artery, and obtained pressure tracings in each chamber. We observed the superior vena cava (SVC) pressure to be markedly elevated, with a gradient of 11 mm Hg over the right atrial (RA) pressure. Furthermore, the RA pressure was 33 mm Hg higher than the RV diastolic pressure. Table 1 displays the hemodynamic measurements observed during right-sided heart catheterization, and Figures 1A to 1D, along with Videos 1, 2, and 3, display the diagnostic angiogram and percutaneous intervention that were performed.

**Table 1:** Hemodynamic Measurements

	Preintervention (mm Hg)	Postintervention (mm Hg)
Blood pressure	95/52	125/58
SVC	56	25
IVC	37	27
RA	45	25
RV	59/12	40/11
PA	61/30	44/22

IVC = inferior vena cava; PA = pulmonary artery; RA = right atrium; RV = right ventricle; SVC = superior vena cava.

## Management

At this time, we believed that the patient required urgent intervention to relieve his obstructive shock, given his hemodynamic instability and high likelihood of imminent death. Surgical consultation was performed by telephone, and the patient was thought to be too unstable for any surgical intervention. We therefore opted for a novel percutaneous approach. With the hemodynamic information that we had obtained, as well as the location of the mass seen on angiography, we identified 2 locations to target for balloon intervention: the SVC-RA junction and the TV annulus. Although we knew that this procedure carried a relatively high risk of serious complications, we believed that the potential benefits vastly outweighed the risks, and we obtained consent from the patient’s substitute decision maker before commencing. A 20-mm NuCLEUS (NuMED) percutaneous catheter balloon was first selected to directly intervene on the lesion and relieve obstruction both at the level of the SVC and across the TV for sequential valvuloplasty. Finally, we performed extensive thrombectomy through which we obtained multiple tan-colored samples for cytologic and tissue analysis. These samples appeared visually to be consistent with tissue, as opposed to thrombus.

**Figure 2:**
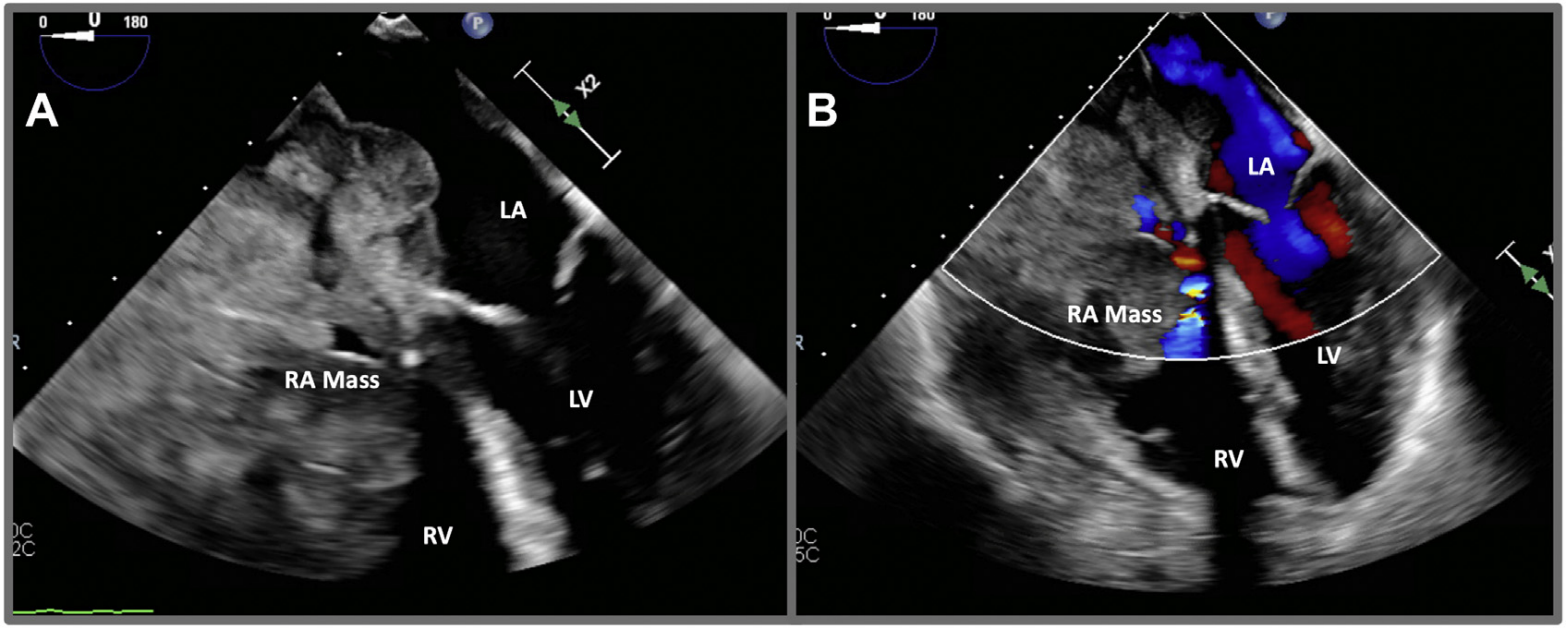
Mass in the RA Seen on TEE **(A)** Transesophageal echocardiography (TEE) imaging displaying a large mass in the right atrium (RA) that was protruding into the right ventricle (RV), as well as across the interatrial septum into the left atrium (LA), indicating a malignant process. **(B)** Accelerated color flow Doppler imaging indicating flow restriction across the tricuspid valve (TV), likely in the location where balloon intervention was performed. LV = left ventricle.

**Figure 3:**
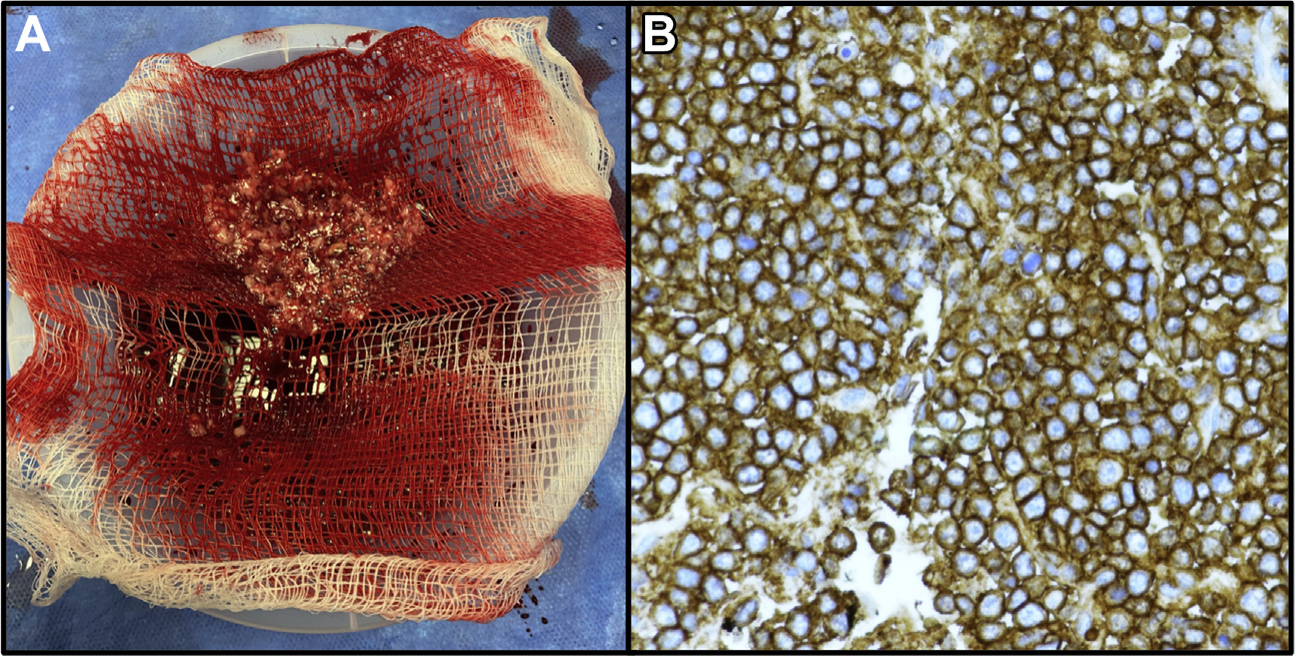
Gross Thrombectomy Specimen and Pathology Slide **(A)** Gross specimen obtained during thrombectomy. **(B)** CD20 immunohistochemically stained slide revealing a mass of large, uniformly CD20^+^ B cells in keeping with high-grade diffuse large B-cell lymphoma.

Following percutaneous intervention, the patient’s hemodynamics improved significantly, and his markers of perfusion normalized. He received an urgent transesophageal echocardiogram, displayed in Figures 2A and 2B and Videos 4 and 5, which demonstrated a tumor with several aggressive and malignant characteristics, including invasion across the interatrial septum into the left atrium. The differential diagnosis of malignant cardiac tumors is displayed in Table 2 (2,3). On day 5 of admission, pathology specimen results were reported as high-grade diffuse large B-cell lymphoma (DLBCL). Figures 3A and 3B displays the gross sample obtained by thrombectomy and the CD20 immunohistochemically stained slide from the pathology laboratory.

**Table 2:** Differential Diagnosis of a Malignant Cardiac Mass

Primary Cardiac Tumors	Metastatic Tumors
Sarcoma Angiosarcoma Rhabdomyosarcoma Leiomyosarcoma Synovial sarcoma Osteosarcoma Fibrosarcoma Myxoid sarcoma Liposarcoma Mesenchymal sarcoma Neurofibrosarcoma Malignant fibrous histiocytoma	Lung cancer Breast cancer Esophageal cancer Sarcoma Lymphoma Leukemia Renal cell carcinoma Melanoma Other less common metastases
Primary cardiac lymphoma	
Primary mesothelioma	

Data from Zipes et al (2) and Tyebally et al (3).

## Discussion

We present a case documenting the use of balloon catheter intervention as a temporizing measure to alleviate de novo obstructive shock state related to a tumor obstructing RV inflow. Obstructive shock encompasses a heterogenous group of processes, and astute clinical judgment is often necessary to discern the underlying cause. Location-specific hemodynamic observations in the catheterization laboratory indisputably assisted in the identification of the obstructive anatomy and allowed for precise percutaneous intervention despite the lack of cross-sectional imaging. Furthermore, aspiration catheter instrumentation facilitated a rapid and definitive oncologic diagnosis by direct tumor and cytologic sampling. Currently, the use of modified interventional techniques such as vacuum-assisted thrombectomy is evolving as a potential new method of intracardiac mass removal and sampling (4). These procedures serve as alternatives to open surgical or interventional radiology-guided biopsies that require considerable resource use and pose potential risk to the patient.

Our case also highlights several key features of cardiac lymphomas, which account for only 1% of all primary cardiac tumors (5). Characteristically, primary cardiac lymphomas can involve direct invasion of the pericardium, right-sided cardiac chambers, and systemic venous great vessels. Mechanical sequelae of tumor invasion may manifest as cardiac tamponade, functional tricuspid stenosis, IVC or SVC syndrome, as well as pulmonary tumor emboli (6). Few cases of cardiac lymphomas complicated by acute hemodynamic failure, from either direct tumor obstruction or tumor emboli, have been noted in published reports. Emergency cardiac surgery has been the upfront treatment strategy in several of these cases (7,8). However, our patient’s profound hemodynamic instability necessitated an innovative percutaneous approach because surgery would have carried prohibitive risk. This unique intervention allowed a period of hemodynamic stability during which time the diagnosis was made and definitive management initiated.

## Follow-Up

Following multidisciplinary case rounds, we concluded that tumor debulking with chemotherapy would carry less risk than surgical debulking and was likely to be effective, given the chemotherapy-responsive nature of DLBCL. The patient was therefore transferred to an intensive care unit at a regional cancer center, and the malignant hematology consultant began treatment that evening with cyclophosphamide, dexamethasone, and rituximab. Unfortunately, 7 days following admission to hospital, the patient went into rapid refractory circulatory collapse and died of his illness. No autopsy was requested. Although we do not have proof of this, we assume that his death was the result of recurrent obstructive shock.

## Conclusions

This case demonstrates the use of a unique intervention in the resuscitation of a patient with obstructive shock and highlights the importance of both astute clinical evaluation and hemodynamic interpretation in the management of this rare presentation.

## Funding Support and Author Disclosures

The authors have reported that they have no relationships relevant to the contents of this paper to disclose.

## Appendix

Supplemental Video 1 Preintervention Angiographic Image of RV. The catheter is seen injecting dye at the superior vena cava–right atrial junction, with restricted filling of the right atrium and RV secondary to tumor obstruction.

Supplemental Video 2 Angiographic Image of Intervention to the TV Annulus. A 20-mm NuCLEUS (NuMED) percutaneous catheter balloon is seen intervening at the level of the TV annulus.

Supplemental Video 3 Postintervention Angiographic Image of RV. The catheter is seen injecting dye at the SVC–RA junction, with significantly improved filling of the right atrium and RV following percutaneous intervention.

Supplemental Video 4 TEE Image of Intracardiac Tumor. Midesophageal 4-chamber TEE image displaying a large obstructive tumor in the RA traversing the TV annulus into the RV.

Supplemental Video 5 TEE Color Doppler Image of Intracardiac Tumor. Midesophageal 4-chamber TEE image displaying a large obstructive tumor in the RA with color Doppler signal indicating accelerated flow across the TV related to obstructive physiology.
